# Antimicrobial Growth Promoters and *Salmonella* spp., *Campylobacter* spp. in Poultry and Swine, Denmark

**DOI:** 10.3201/eid0904.020325

**Published:** 2003-04

**Authors:** Mary C. Evans, Henrik C. Wegener

**Affiliations:** * Danish Veterinary Institute, Copenhagen, Denmark

**Keywords:** Antimicrobial growth promoters, Salmonella, Campylobacter, poultry, swine, dispatch

## Abstract

The use of antimicrobial growth promoters in Danish food animal production was discontinued in 1998. Contrary to concerns that pathogen load would increase; we found a significant decrease in *Salmonella* in broilers before and after slaughter of swine and pork and no change in the prevalence of *Campylobacter* in broilers.

Antimicrobial growth promoters are antimicrobial drugs added to animal feed to enhance growth and improve feed efficiency of food animals. In the United States, the use of antimicrobial growth promoters also includes elements of prophylaxis, which are not allowed in Europe. Antimicrobial growth promoters have been widely used in Danish food animal production since the 1970s. On February 15, 1998, the Danish cattle and broiler industries, reacting to consumer concerns over food safety, voluntarily stopped the use of all antimicrobial growth promoters. The pig industry stopped using the growth promoters in pigs over 35 kg; all use was phased out in 1999.

Despite concerns that no longer using the growth promoters would have a wide range of negative effects (e.g., an increase in disease and death, poor growth rates, increased feed consumption, increased fecal shedding, and enhanced shedding or carriage of foodborne pathogens such as *Salmonella* or *Campylobacter*), producers in the broiler and pig industries discontinued the use. Studies have shown that antimicrobial drugs reduce part of the intestinal flora while potentially decreasing pathogen shedding (*1*–*3*). For these reasons, producers believed that removing antimicrobial growth promoters could cause human pathogenic intestinal bacteria in food animals to increase. Another concern was that increased fecal shedding could lead to contamination of carcasses at slaughter and increased risk for foodborne infection in humans.

Our study examines the effect of discontinued use of antimicrobial growth promoters on the prevalence of *Salmonella* in Danish broiler flocks, chickens after slaughter, swine herds, and pork end products. We also examine the effect on *Campylobacter* in Danish broiler flocks.

## The Study

Data for this analysis were obtained from routine monitoring programs in Danish broilers and swine. Approximately 450,000 broiler and chicken samples and 830,000 swine and pork samples are tested each year. A detailed description of sample collection methods and numbers of samples is available (*4*).

Broiler flocks have been tested for *Salmonella* since 1989 and for *Campylobacter* since the end of 1995. Initially, flocks were tested for *Salmonella* by the collection of 16 fecal samples; however, since June 2000, five “sock samples” (Samples were taken by placing a sock over the collector’s shoes. The collector walks through the poultry house; the socks absorb fecal samples) have been collected. Antemortem samples are obtained 2–3 weeks before slaughter by collecting five pairs of sock samples per flock (*4*). Before November 2000, postmortem *Salmonella* sampling was conducted by examination of five pooled swab samples each consisting of 10 neck-skin samples per flock. After November 2000, postmortem sampling came from batches of poultry parts. Because of this change, we excluded postmortem data (after November 2000) from analysis. Broiler flocks are monitored for *Campylobacter* by examination of cloacal swabs from 10 birds per flock or batch at slaughter.

Since June 1995, swine herds have been continuously monitored for *Salmonella* by serologic testing of “meat juice” (10g of muscle tissue are collected from the neck, diaphragm or tenderloin of the animal. This sample is placed into a container consisting of an upper coffee-filter like part to hold the meat and a lower tube-like part. The container is frozen overnight at –20°C and subsequently allowed to thaw at 4°C for 24 hours, causing release of the meat juice into the lower part of the tube. This juice is then tested for *Salmonella* antibodies) samples from each herd producing >100 finishers (pigs 30–50 kg) per year. In July 2001, this requirement changed to herds producing >200 finishers per year. The number of samples taken is dependent on herd size. Based on serologic results, herds are placed into categories. Level 1 herds have no or few seroreactors (animals that test positive for *Salmonella*), and no intervention is required. Level 2 herds have a higher proportion of seroreactors, and the herd owner must seek advice on reducing the prevalence of *Salmonella*. Level 3 herds have a large proportion of seroreactors, and the herd owner must seek advice and slaughter under special hygienic conditions.

Since July 1993, *Salmonella* in pork has been measured by monthly samples of cuts of meat from slaughterhouses. The number of samples depends on the number of animals slaughtered. In January 2001, the sampling method changed to carcass swabs. Consequently, data from 2001 were not included in this analysis (*4*).

Data were divided into two periods: before (P1) and after (P2) withdrawal of antimicrobial growth promoters. To account for factors such as seasonality, equal time periods were used for comparison; however, periods varied from 12 to 36 months, depending on availability of data. Although the voluntary discontinuation on purchasing antimicrobial growth promoter-containing feed for broilers was initiated on February 15, depletion of feed stocks may have taken several weeks. To account for this and for the uneven withdrawal of antimicrobial growth promoters in pigs, data from 1998 (broilers) and 1998–1999 (swine) were excluded from analysis. The resulting time periods are found in Table 1.

**Table 1:** Description of time intervals used for data analysis, by species and pathogen^a^

Species	Pathogen	Period 1 (P1)	Period 2 (P2)
Broilers	*Salmonella* (antemortem )	Jan 1995–Dec 1997	Jan 1999–Dec 2001
	*Salmonella* (postmortem)	Jan 1996–Oct 1997	Jan 1999–Oct 2000
	*Campylobacter*	Jan 1996–Dec 1997	Jan 1999–Dec 2000
Swine	*Salmonella*	Jan 1996–Dec 1997	Jan 2000–Dec 2001
	*Salmonella* (pork)	Jan 1997–Dec 1997	Jan 2000–Dec 2000

^a^Period 1 and 2 refer to the time periods before (P1) and after (P2) the withdrawal of antimicrobial growth promoters.

For this analysis, the mean prevalence of positive flocks (broilers), the percentage of herds classified as level 2 or level 3 (swine herds), and the percentage of positive samples (pork) were obtained by month for P1 and P2. Using these data, the mean prevalence for P1 and P2 were calculated and differences in means were evaluated by a t test by using SAS version 8.0 (SAS Institute, Inc., Cary, NC). The prevalence of *Salmonella* and *Campylobacter* in broilers and swine between 1995 and 2001 is shown in the Figure.

**Figure:**
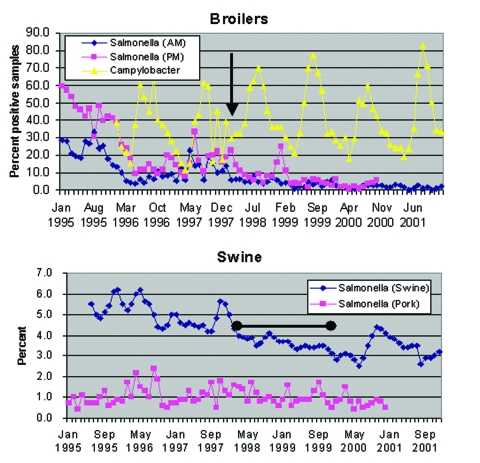
Prevalence of *Salmonella* and *Campylobacter* in Danish broiler flocks, chicken meat, swine herds, and pork products, 1995-2001.The arrow indicates February 15, 1998 the date of the voluntary stop of AGP use in broilers. The bar indicates the time period during which antimicrobial growth promoters were withdrawn from use in swine herds.

The mean percentage of broiler flocks testing positive for *Salmonella* during P1 (before withdrawal) was 14.4% (range 3.7% to 33.6%) for antemortem examination and 17% (range 8.1% to 38.8%) for postmortem (Table 2) P2 samples averaged 2.4% (range 0.2% to 5.8%) and 4.9% (range 0.7% to 24.9%), respectively. A comparison of means showed that the prevalence of *Salmonella* was significantly lower in the period following withdrawal of antimicrobial growth promoters for both antemortem (p<0.0001) and postmortem samples (p<0.0001). The average percentage of broiler flocks testing positive for *Campylobacter* during P1 was 35.3% (11.4% to 64.%8) and P2 was 40.8 (18% to 77%). No statistical difference existed in the mean prevalence between the two periods (p=0.2470).

**Table 2:** Comparison of the average prevalence of *Salmonella* and *Campylobacter* in broiler flocks, chicken meat, swine herds, and pork products before and after withdrawal of antimicrobials for use as growth promoters

	Period 1^a^ mean (range)	Period 1 standard deviation	Period 2^b^ mean (range)	Period 2 standard deviation	p value
*Salmonella*					
Broilers (antemortem)	14.4 (3.7–33.6)	8.3902	2.4 (0.2–5.8)	1.3215	<0.0001
Broilers (postmortem)	17.0 (8.1–38.8)	7.9125	4.9 (0.7–24.9)	5.0493	<0.0001
Swine	5.0 (4.2–6.2)	0.5904	3.3 (2.5–4.4)	0.5347	<0.0001
Pork	1.1 (0.5–1.8)	0.3895	0.8 (0.4–1.5)	0.2839	0.0290
*Campylobacter*					
Broilers (antemortem)	35.3 (11.4–64.8)	16.6290	40.8 (18.0–77.0)	16.1185	0.2470

^a^Before withdrawal of antimicrobial growth promoters. ^b^After withdrawal of antimicrobial growth promoters.

The percentage of swine herds classified as level 2 or level 3 during P1 was 5% (4.2% to 6.2%) and 3.3% (2.5% to 4.4%) for P2. A comparison of means showed that the average percentage of swine herds classified as level 2 or 3 was significantly lower in the period following the withdrawal of antimicrobial growth promoters (p<0.0001). The percentage of *Salmonella* isolated from fresh pork samples dropped from 1.1% (0.5% to 1.8%) in P1 to 0.8% (0.4% to 1.5%) in P2. Although the change is small, the period following the withdrawal of antimicrobial growth promoters was significantly lower (p=0.0290).

## Conclusions

Contrary to concerns that withdrawal of antimicrobial growth promoters would cause an increase in pathogen load, we found a decrease in *Salmonella* prevalence in broilers, chicken, swine, and pork and no change in the prevalence of *Campylobacter* in broilers. Previous studies on this topic have shown mixed results. Two observational studies found that penicillin given to swine increased total bacterial and *Enterobacteriaceae* counts (*5*,*6*). Other experiments found that avoparcin increased *Salmonella* shedding in broilers and excretion rates had a dose-response effect with increasing concentrations of avoparcin (*7*,*8*). A series of experiments in broilers showed that avoparcin, nitrovin, tylosin, flavomycin, and lincomycin caused increased shedding of *Salmonella* in most experiments, while virginiamycin and bacitracin had little or no effect and sodium arsenilate decreased shedding (*9*–*11*). Holmberg et al. found that both avoparcin and monensin reduced shedding of *S.* Infantis in broilers but a combination of the two increased shedding (*12*). Bolder et al. showed that flavophospholipol and salinomycin decreased *Salmonella* shedding in broilers but had no significant effect on the shedding of *C. jejuni* (*13*).

Our study is unique because we included a large sample of animals under natural conditions, included both animals and products, and examined the combined effect of many antimicrobial growth promoters. However, several factors should be kept in mind when interpreting the results. First, our analysis cannot elucidate the impact that withdrawal of an individual antimicrobial growth promoter had on a particular pathogen or in a particular species. In addition, since avoparcin was withdrawn in 1995, any immediate effects seen from its discontinued use will be demonstrated in P1 of our study instead of P2. Despite a change in sampling methods for broilers in June 2000 and swine herds in July 2001, these data were included in analysis. Both changes increased the sensitivity of sampling, in theory leading to a higher prevalence. Since this change occurred during P2, it would tend to bias our results toward the null; thus, including these samples gives our study a more conservative result. Finally, our study only describes the prevalence of *Salmonella* and *Campylobacter* after the withdrawal of antimicrobial growth promoters. Effects such as productivity, changes in therapeutic antimicrobial drug use and economic impact are described in another study (*14*).

Our findings only show a temporal relationship between withdrawal and reduction, and one should be cautious not to infer causality. The fact that the decrease was seen before and during the use of antimicrobial growth promoters suggests that other factors play a role. The most obvious of these factors is the effect of the ongoing surveillance and control programs in food-producing animals. Programs in broilers and swine, described each year in the Annual Report on Zoonosis (*4*), have been in effect since the late 1980s and mid-1990s and have made a substantial impact on reducing the prevalence of *Salmonella* in primary food production. What is clearly shown from this analysis is that *Salmonella* and *Campylobacter* rates have not increased in food animal carriers since antimicrobial growth promoters were withdrawn in 1998. This finding, combined with evidence that the withdrawal has taken place without remarkably noticeable effects on the productivity in broilers (*15*) and swine, is of particular importance in light of the emerging problem of antimicrobial drug-resistant human pathogenic organisms, which are associated with the use of antimicrobial growth promoters.
